# A novel endoscopic lithotripsy technique for a huge common bile duct stone: endoscopic snare lithotripsy

**DOI:** 10.1055/a-2663-8259

**Published:** 2025-08-22

**Authors:** Hidekazu Tanaka, Mamoru Takenaka, Yasuhiro Masuta, Kosuke Minaga, Koichiro Kawano, Masatoshi Kudo

**Affiliations:** 1 Department of Gastroenterology and Hepatology, Kindai University Faculty of Medicine, Osaka-Sayama, Japan; 2 13865 Department of Gastroenterology, Hyogo Prefectural Awaji Medical Center, Sumoto, Japan

Recently, large common bile duct (CBD) stones have been treated with endoscopic papillary large balloon dilation (EPLBD) 1 2 or electronic hydraulic lithotripsy (EHL) 3 4 ; however, difficult-to-treat cases remain 5 . Here, we report a case in which a novel lithotripsy method using a snare was successfully used to treat a large stone occupying the entire CBD lumen.

An 85-year-old man with recurrent biliary obstruction secondary to obstructive cholangitis caused by a large CBD stone was referred to our hospital for stone removal.

Cholangiography confirmed the presence of a stone occupying the CBD lumen, and the end of the stone was delivered from the duodenal papilla ( Fig. 1 ). Since neither EPLBD nor EHL could be performed, we attempted to grasp the delivered part with grasping forceps, but the stone just broke up gradually, and the procedure was unsuccessful ( Fig. 2 ).

**Fig. 1:**
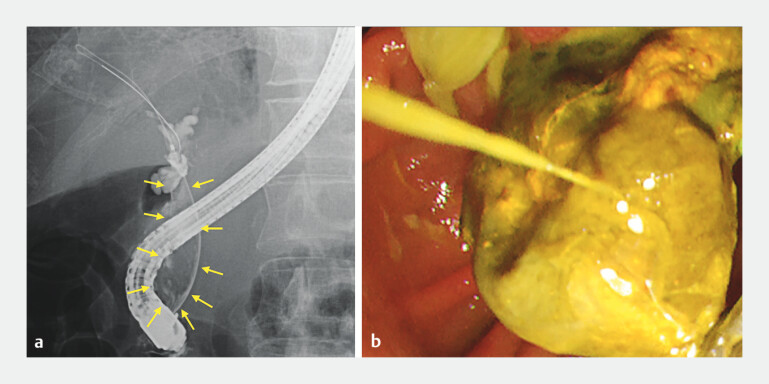
**a** Cholangiography confirms a stone occupying the common bile duct lumen (yellow arrow). **b** The end of the stone is delivered from the duodenal papilla.

**Fig. 2:**
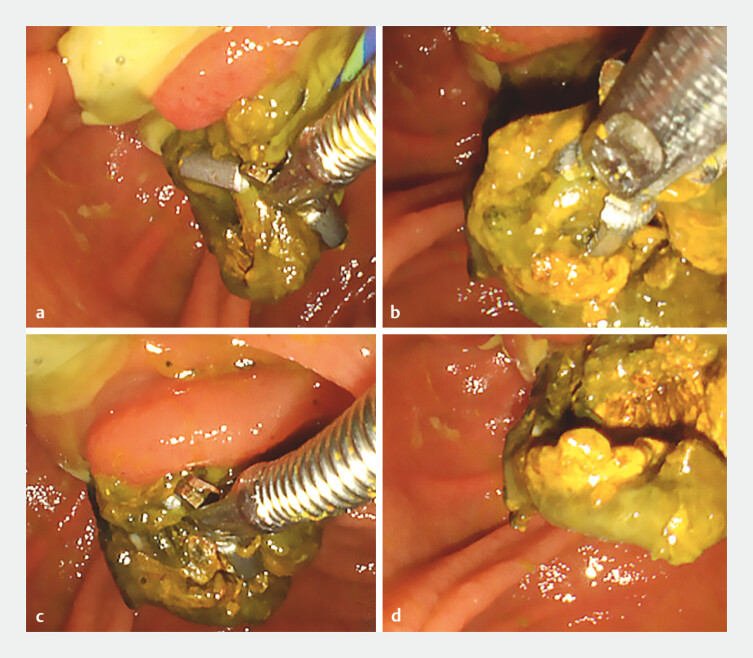
We attempted to grasp the delivered part with grasping forceps, but the stone just broke gradually and the procedure was unsuccessful.

However, it was possible to grasp the stone without breaking it up using a snare (Snare Master Plus, 15 mm; Olympus, Tokyo, Japan) and to perform electronic stone cutting with snaring ( Fig. 3 ).

**Fig. 3:**
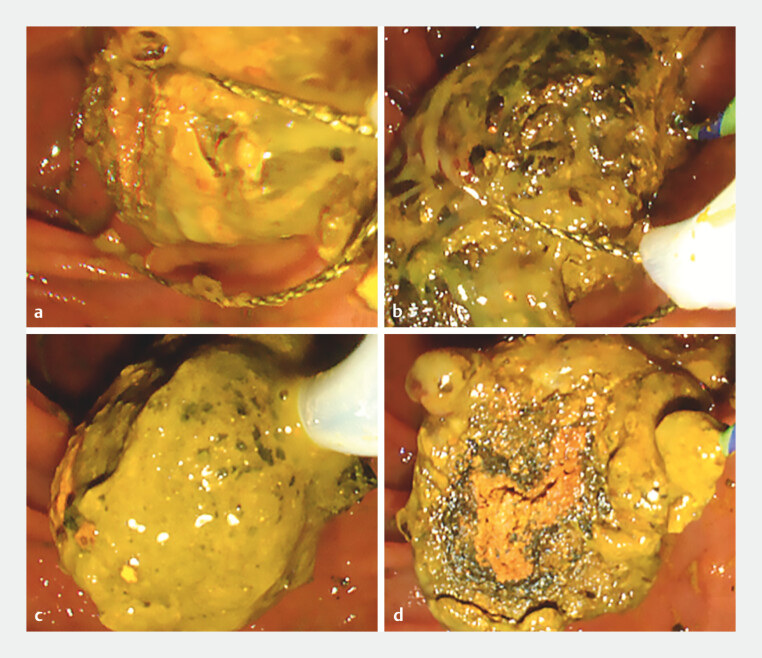
The stone was grasped without breaking using a snare, and electronic stone cutting with snaring was possible.

We attempted to remove the stone by repeatedly pulling it out to a length at which it could not be pulled out any further, cutting it with the snare, leaving the part near the papilla that was being grasped, grasping it again, pulling it out, and repeating the cutting ( Fig. 4 ). By repeating this process several times, we completely removed the large stone ( Fig. 5 , Video 1 ).

**Fig. 4:**
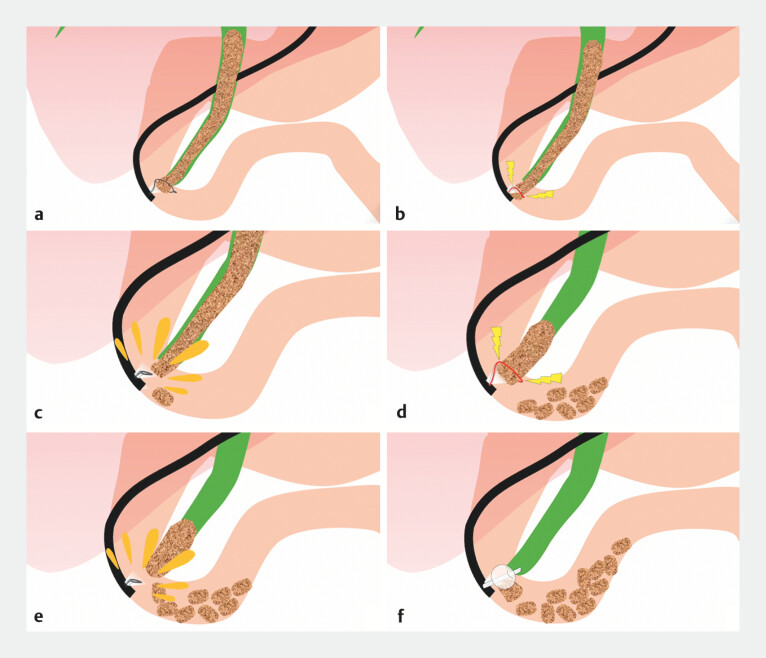
We removed the stone by repeatedly pulling it out to a length at which it could not be pulled out any further, cutting it with a snare, and leaving the part that is being grasped near the papilla.

**Fig. 5:**
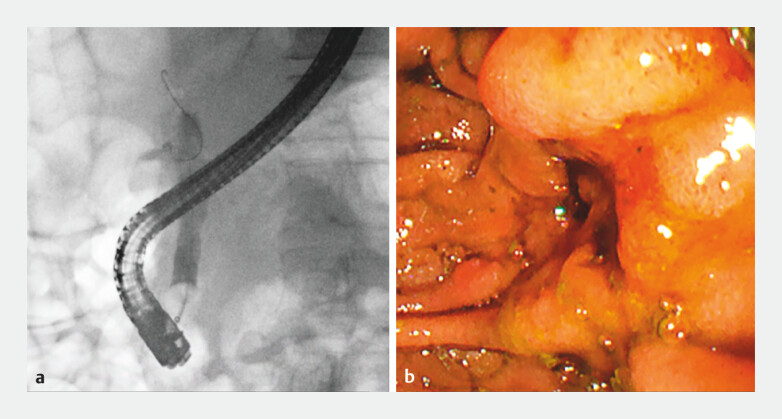
By repeating endoscopic snare lithotripsy several times, we completely removed the stone.

This video introduces a novel endoscopic snare lithotripsy technique for treating large common bile duct stones.Video 1

This novel endoscopic snare lithotripsy may be a valuable option for removing difficult-to-treat large stones.

Endoscopy_UCTN_Code_TTT_1AR_2AH

## Notes

Endoscopy E-Videos https://eref.thieme.de/e-videos
*E-Videos* is an open access online section of the journal *Endoscopy* , reporting on interesting cases and new techniques in gastroenterological endoscopy. All papers include a high-quality video and are published with a Creative Commons CC-BY license. Endoscopy E-Videos qualify for HINARI discounts and waivers and eligibility is automatically checked during the submission process. We grant 100% waivers to articles whose corresponding authors are based in Group A countries and 50% waivers to those who are based in Group B countries as classified by Research4Life (see: https://www.research4life.org/access/eligibility/ ). This section has its own submission website at https://mc.manuscriptcentral.com/e-videos .
